# Metabolomics Analysis as a Tool to Measure Cobalt Neurotoxicity: An In Vitro Validation

**DOI:** 10.3390/metabo13060698

**Published:** 2023-05-27

**Authors:** Ibrahim M. Alanazi, Abdullah R. Alzahrani, Torki A. Zughaibi, Ahmed I. Al-Asmari, Shams Tabrez, Catherine Henderson, David Watson, Mary Helen Grant

**Affiliations:** 1 Department of Pharmacology and Toxicology, Faculty of Medicine, Umm Al-Qura University, Al-Abidiyah, Makkah 21955, Saudi Arabia; 2 Department of Medical Laboratory Sciences, Faculty of Applied Medical Sciences, King Abdulaziz University, Jeddah 21589, Saudi Arabia; 3 King Fahd Medical Research Center, King Abdulaziz University, Jeddah 21589, Saudi Arabia; 4 Laboratory Department, King Abdul-Aziz Hospital, Ministry of Health, Jeddah 22421, Saudi Arabia; 5 Toxicology and Forensic Science Unit, King Fahd Medical Research Center, King Abdulaziz University, Jeddah 21589, Saudi Arabia; 6 Department of Biomedical Engineering, University of Strathclyde, Glasgow G4 0NW, UK; 7 Strathclyde Institute of Pharmacy & Biomedical Sciences, University of Strathclyde, Glasgow G4 0RE, UK

**Keywords:** cobalt, neurotoxicity, in vitro, mass spectroscopy, metabolomics

## Abstract

In this study, cobalt neurotoxicity was investigated in human astrocytoma and neuroblastoma (SH-SY5Y) cells using proliferation assays coupled with LC–MS-based metabolomics and transcriptomics techniques. Cells were treated with a range of cobalt concentrations between 0 and 200 µM. The 3-(4,5-dimethylthiazol-2-yl)-2,5-diphenyltetrazolium bromide (MTT) assay revealed cobalt cytotoxicity and decreased cell metabolism in a dose and time-dependent manner was observed by metabolomics analysis, in both cell lines. Metabolomic analysis also revealed several altered metabolites particularly those related to DNA deamination and methylation pathways. One of the increased metabolites was uracil which can be generated from DNA deamination or fragmentation of RNA. To investigate the origin of uracil, genomic DNA was isolated and analyzed by LC–MS. Interestingly, the source of uracil, which is uridine, increased significantly in the DNA of both cell lines. Additionally, the results of the qRT-PCR showed an increase in the expression of five genes Mlh1, Sirt2, MeCP2, UNG, and TDG in both cell lines. These genes are related to DNA strand breakage, hypoxia, methylation, and base excision repair. Overall, metabolomic analysis helped reveal the changes induced by cobalt in human neuronal-derived cell lines. These findings could unravel the effect of cobalt on the human brain.

## 1. Introduction

Cobalt is an essential metal that is found in cobalamin (vitamin B12) in the human body [1]. Co^2+^ is proposed to produce hydroxyl radicals, to up-regulate hypoxia-inducible factors, and to have Ca^2+^ and Fe^2+^ antagonism properties [2]. There are over a million workers who deal with and are at risk of exposure to toxic concentrations of cobalt [3]. It is used in many industries such as rechargeable battery manufacture, metal alloy production, and in magnetics due to its physical properties (improved conductivity, low corrosiveness, elevated melting point, and magnetic function). It is also used in steel manufacturing, the diamond industry, dying, and pigment production [4]. People exposed to cobalt through inhalation at work have encountered some effects on their nervous system such as hearing loss, lack in visual ability and memory loss [5,6]. When rats and mice inhaled cobalt sulfate (19 mg/m^3^) for 16 days, they developed congestion in their brain blood vessels [7,8].

Metabolomics has been widely used to investigate mechanisms of toxicant action in human cells [9]. Metabolomic analysis measures the full metabolome and illustrates the change in the biochemical pathways after exposure to toxic compounds (Robertson, 2005). Metabolic profiling has been reported for multiple drugs exhibiting the change in the metabolome and the biochemical pathways [10]. The most common approach to conducting metabolomics analysis is to apply mass spectrometry (MS), or nuclear magnetic resonance (NMR). MS metabolomics is the method of choice for biomarker discovery because of its high sensitivity and specificity [11]. Liquid chromatography–mass spectrometry (LC–MS) is a combined technique for the separation of different chemicals by liquid chromatography (or high-performance liquid chromatography) with the detection technique of mass spectrometry [12,13].

To our knowledge, there have been no studies on the effect of cobalt in cell culture-based studies, especially using human neuronal cells. The in vitro cell culture model was used to minimize variation during experiments and to study them under precise and simply evaluated environments. It is widely agreed that it is impossible for in vitro culture to simulate whole animal studies; however, cell culture studies are widely used to study chemical metabolism and toxicity in vitro. Since little is known about the mode of action of cobalt in neuronal cells, the current study aimed to investigate the mode of action of cobalt in the human astrocytoma (U373) and neuroblastoma (SH-SY5Y) cell lines using multiple toxicology and metabolomics analysis methods.

## 2. Materials and Methods

Complete Dulbecco’s Modified Eagle Medium (DMEM) media containing 10% Foetal Bovine Serum (FBS) (Biosera, Metro Manila, Philippines), 50 µg/ mL streptomycin/50 IU/ mL penicillin mixture, and 1% non-essential amino acids (NEAA) was used to grow U-373 cells. On the other hand, complete DMEM/F12 media was used to grow SH-SY5Y cells.

### 2.1. Cell Lines Culture as Monolayer

Human astrocytoma (U373), and neuroblastoma (SH-SY5Y) cells were purchased from the European Collection of Cell Cultures (catalogues number 08061901, and 94030304, respectively). The studied cell lines were seeded at 5 × 10^4^ cells/well in 1 mL media in 24-well plates for metabolism and metabolomics analysis, and in 200 μL of medium in a 96-well plate for MTT, ROS, and qRT-PCR assays.

### 2.2. Dimethylthiazolyl Diphenyltetrazolium Bromide (MTT) Assay

MTT assay is based on the ability of NADPH-dependent cellular oxidoreductase enzymes present in the viable cells to reduce the tetrazolium dye (MTT) to formazan [14,15]. A total of 10 mM of MTT solution was prepared in PBS and filtered through a 0.2 μM filter. A 50 μL volume of the solution was added to each well when the incubation was complete. A 96-well plate was incubated at 37 °C in the presence of 5% CO_2_ air for 4 h. MTT solution was removed after incubation and 200 μL of DMSO was added to each well. The solution in each well was mixed to give an even color and transferred to a 1 mL cuvette. Absorbance was measured at 540 nm by plate reader spectrophotometer (Multiskan GO, Thermo Scientific, Waltham, MA, USA). The mean of 6 replicates and *n* = 3 experiments were calculated by adding all absorbance values and dividing them by the number of replicates (6). The percentage mean was then calculated for each concentration by dividing the mean of the concentration by the mean of the control and multiplying the results by 100.

### 2.3. Reactive Oxygen Species (ROS)

Measurement of ROS activity was performed using carboxy-H_2_DCFDA dye (Invitrogen, Waltham, MA, USA, UK C400, Lot number 28351W) as described earlier [16,17]. Cells were seeded at 5 × 10^4^ cells/ mL and incubated at 37 °C and 5% CO_2_/air for two days. The cells were then treated with a range of cobalt concentrations (0, 100, 150, and 200 μM) for two periods of time (4 and 24 h). Then the medium was removed from all wells, and the cells were washed twice with DPBS to remove any traces of media from the wells. The addition of 200 μL of carboxy-H2DCFDA at 25 μM in DPBS followed the washing step. The culture was incubated in the dark at 37 °C and 5% CO_2_ air for 30 min. Cells were washed twice with DPBS after incubation. Cells were examined by microscope (ZOE Fluorescent Cell Imager, BIO-RAD, Singapore) and pictures were taken immediately. Finally, triton X-100 (1 mL at 0.1% (*v*/*v*)) was added to the wells and they were incubated in the dark for 20 min at room temperature. Measurement of the fluorescence was immediately undertaken by a spectrofluorophotometer (RF-5001PC, Shimadzu, Kyoto, Japan) at 495 nm excitation wavelength and 525 nm emission wavelength in a 1 mL cuvette.

### 2.4. DNA and RNA Isolation

DNA and RNA were isolated from U-373 and SH-SY5Y cells according to the procedures in the isolation kit Illustra triplePrep kit (GE Healthcare, Amersham, UK) for nuclear DNA and total RNA isolation. Cells were grown in 25 cm flasks at 5 × 10^4^ seeding density in three replicates for each cobalt concentration and controls. Flasks were left overnight to allow cells to attach to the flask surface. Cobalt at 0, 100, 150, and 200 μM concentrations in a medium was added to the cells after removing the old medium. Then the flasks were incubated at 37 °C and 5% CO_2_ air for 72 h. Flasks were washed with cold DPBS. The cells in the control flasks were treated with 1 mL of lysis buffer (containing 3.5 μL 2-mercaptoethanol) from the Illustra triplePrep kit. The flasks were agitated until they gave a cloudy solution. Lysates were transferred to Eppendorf tubes to either continue the extraction or stored at −80 °C until ready to extract the nucleic acids. Each lysate was used to isolate DNA and RNA using the procedure of the Illustra triplePrep kit. Both isolated nucleic acids were stored at −80 °C.

### 2.5. DNA Digestion

Isolated DNA samples (20 μg) were incubated overnight in a hydrolysis solution (50) μL that consisted of 100 mM NaCl, 20 mM Tris pH 7.9, 20 mM MgCl_2_, 80 U/mL alkaline phosphatase, 600 mU/mL phosphodiesterase, 1000 U/mL benzonase, 36 μg/mL EHNA hydrochloride, and 2.7 mM deferoxamine. The hydrolyzed solution was diluted in acetonitrile by a 1:4 dilution factor.

### 2.6. Quantitative Real-Time PCR (qRT-PCR)

A PowerUp^TM^ SYBR^TM^ Grean Master Mix (Thermo-Fisher Scientific, Waltham, MA, USA) and a StepOnePlus^TM^ RT-PCR system (Applied Biosystems; Warrington, UK) were used. Primers were designed by the use of the National Center for Biotechnology Information website [18]. The design aimed to yield a length of 100–150 bp amplicon to cover the exons to avoid any amplification of genomic DNA. The designed primers are illustrated in Table 1. RNA and DNA integrity and quantity were assessed using a Nanodrop 2000 spectrophotometer (Thermo Scientific, Waltham, MA, USA) before analysis. The complementary DNA (cDNA) was synthesized of 4 μg of RNA using a Tetro cDNA Synthesis kit (Bioline Reagents, London, UK) and Random Hexamer (500 ng/μL, Bioline Reagents, London, UK).

### 2.7. Extraction of Cell Lysates for Metabolomics

The medium was removed from wells and the cells were washed with 0.5 mL of pre-warmed PBS (37 °C) twice. A 200 μL volume of pre-cooled extraction solution (50% of methanol, 30% of acetonitrile, and 20% water) was added to each well to lyse cells. Cells were scraped off using a small metallic spatula. The extracted cell solution was transferred to 0.5 mL Eppendorf vials. Samples were then shaken at 4 °C for 12 min. Samples were centrifuged at 0 °C and 13,000 rpm for 10 min. The supernatant was transferred into glass vials and stored at −80 °C until analyzed with LC–MS.

### 2.8. Metabolomics

Analysis for metabolomics was carried out by liquid chromatography–mass spectrometry (LC–MS) technology using a Thermo Exactive Orbitrap instrument (Thermo-Fisher Corporation, Hemel Hempstead, UK) as described previously (Kamleh et al., 2009 [18]). Detection of negative and positive ions was used in sample analysis. The scanned mass range was 75–1200 *m*/*z*. The temperature of the capillary was 250 °C, and +4.0 kV spray voltage was used in positive ion and −4.0 kV in negative ion, sheath gas flow was 50 auxiliary gas flow was 17 (arbitrary units). The UltiMate 3000 LC–MS system was run using Xcalibur Ver. 2.2 (Thermo-Fisher Corporation, Hemel Hempstead, UK). The HPLC method used a binary gradient with an injection volume of 10 μL. Solvent A was acetonitrile and solvent B was ammonium carbonate 20 mM; the flow rate was 0.3 mL min^−1^. The column used was a ZIC^®^-pHILIC (150 mm × 4.6 mm i.d., particle size: 5 μm) and was fitted with a ZIC^®^-pHILIC guard column (HiChrom Limited, Reading, UK) at room temperature. Data processing was carried out using Mzmine 2.21 [19], SIMCA-P software v.14.1 (Umetrics AB, Umeå, Sweden), and Excel (Microsoft, Redmond, WA, USA) software. Compounds were identified to MSI level 2 (matching of elemental composition to an accurate mass to <3 ppm—also considering likely retention times e.g., lipids and fatty acids would not be expected to strongly retain on the ZICpHILIC column) or MSI level 1. Accurate mass plus matching to retention times (to within ±0.3 min) of authentic standards run on the ZICpHILIC column (Table 1) [20]. Normalization was not performed during analysis as the cells were counted and the metabolites were normalized by adding an equal volume of extraction solution for the same cell number. Positive and negative ion data sets were combined using an in-house programmed Excel file. Databases used for the identification of metabolites were KEGG, Hmdb, Metlin, and Lipid maps.

### 2.9. Statistical Methods

One-way analysis of variance (ANOVA) and Tukey’s post hoc honestly significant difference test were used to statistically analyze our data. For the metabolomic analysis, the *p*-values were calculated by performing the T-test in Excel according to the following equation. *t*-test for metabolite x = (average peak areas of metabolite x in treated samples, average peak areas of metabolite x in control samples, 2, 3). Where x is the metabolite of interest, 2 is a two-tail distribution and 3 is two samples assuming unequal variance. In addition, false discovery rate (FDR)-adjusted *p*-values with Benjamini–Hochberg method were used to avoid false positive data in supplementary Tables S5 and S6. *p* < 0.05 was considered a significant criterion.

## 3. Results

### 3.1. Measurement of MTT Reduction in Neuroblastoma Cells in the Presence of Cobalt

**Figure 1:**
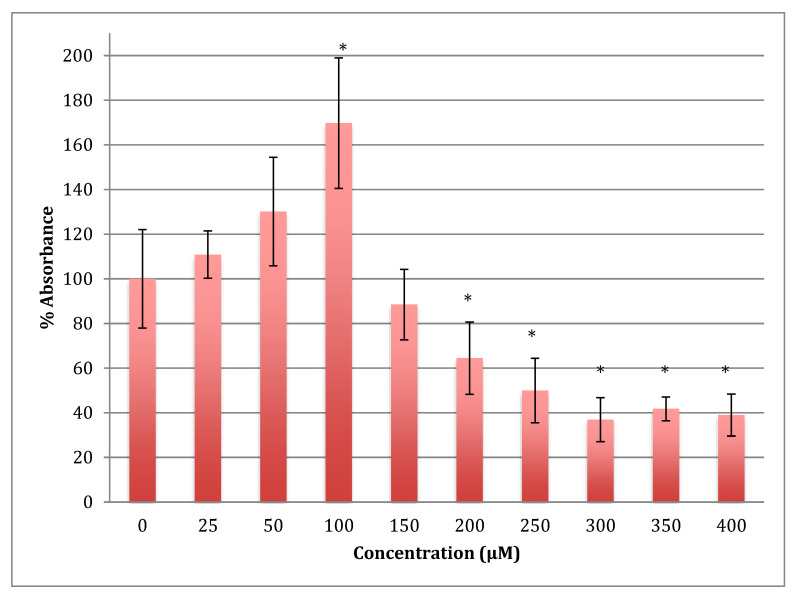
MTT assay measured in neuroblastoma cells at 24 h. Results are percentage values (Mean ± SD, *n* = 3) where 100% corresponds to control values. SD values correspond to an average of 6 wells plate absorbance reading. Cells were treated with cobalt in a culture medium throughout the experiment. Data were analyzed by One-way ANOVA followed by a Tukey test. * Represents significantly different mean values between treatment and control. *p*-value < 0.05.

The MTT results showed an increase in the metabolism in SH-SY5Y cells when treated with cobalt at concentrations 25 and 50 reaching the maximum at 100 μM for 24 h (Figure 1). Beyond this concentration, metabolism fell off with LD_50_ being at 254 ± 17 μM of cobalt.

**Figure 2:**
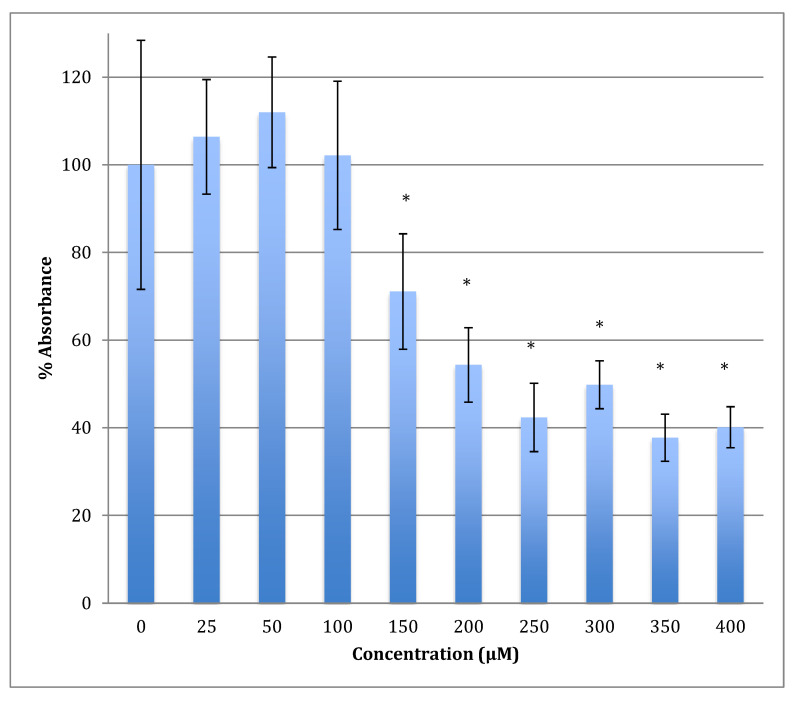
MTT assay measured in neuroblastoma cells at 48 h. Results are percentage values (Mean ± SD, *n* = 3) where 100% corresponds to control values. Cells were treated with cobalt in a culture medium throughout the experiment. Data were analyzed by One-way ANOVA followed by a Tukey test. * Represents significantly different mean values between treatment and control. *p*-value < 0.05.

**Figure 3:**
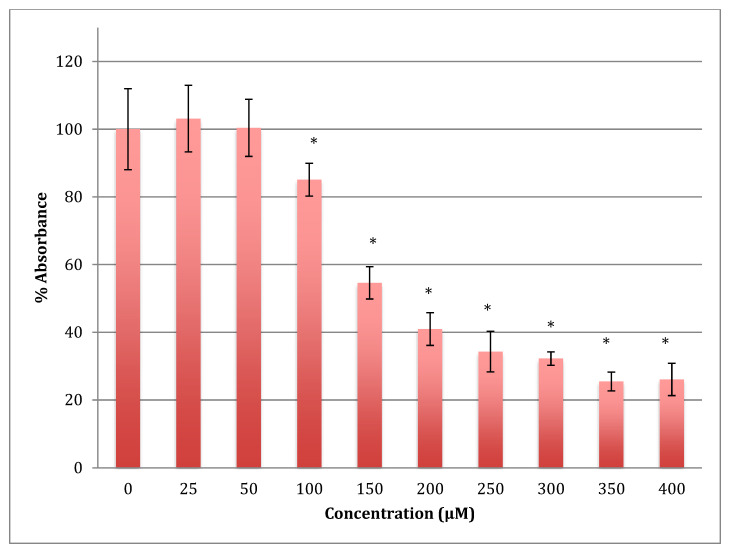
MTT assay measured in neuroblastoma cells at 72 h. Results are percentage values (Mean ± SD, *n* = 3) where 100% corresponds to control values. Cells were treated with cobalt in a culture medium throughout the experiment. Data were analyzed by One-way ANOVA followed by a Tukey test. * Represents significantly different mean values between treatment and control. *p*-value < 0.05.

The time of incubation with cobalt increased to 48 h to find out if the effect changed with time (Figure 2). Increasing the time of incubation did change the metabolism and showed less change in cells treated with lower concentrations of cobalt (25, 50, and 100 μM). There were no significant changes in viability between non-treated (control) cells and cells treated with these concentrations. Though, compared to the same concentrations in a shorter period of incubation (24 h) there was less increase in metabolism indicating that the cells are on their way to lower metabolism which is supported by the 72 h of incubation data (Figure 3). The unchanged metabolism in these low concentrations is also an indicator of the ability of the cell to repair and function properly in these concentrations and with this time of incubation (48 h). However, cells treated with higher concentrations (150–400 μM) of cobalt showed a significant decrease in viability similar to that observed at 24-h incubation. After 48 h the LD_50_ of cobalt was 220 ± 15 μM. Thus, it was proposed that the SH-SY5Y cells could tolerate cobalt treatment up to 100 μM at 48 h incubation. Treatment with cobalt at higher concentrations (>100 μM) would affect the ability of cells to maintain normal metabolism. After 72 h of incubation, the cell metabolism could tolerate cobalt treatment only up to 50 μM, and the LD_50_ of cobalt was 102 ± 14 μM.

### 3.2. Measurement of MTT Reduction in Astrocytoma Cells in the Presence of Cobalt

**Figure 4:**
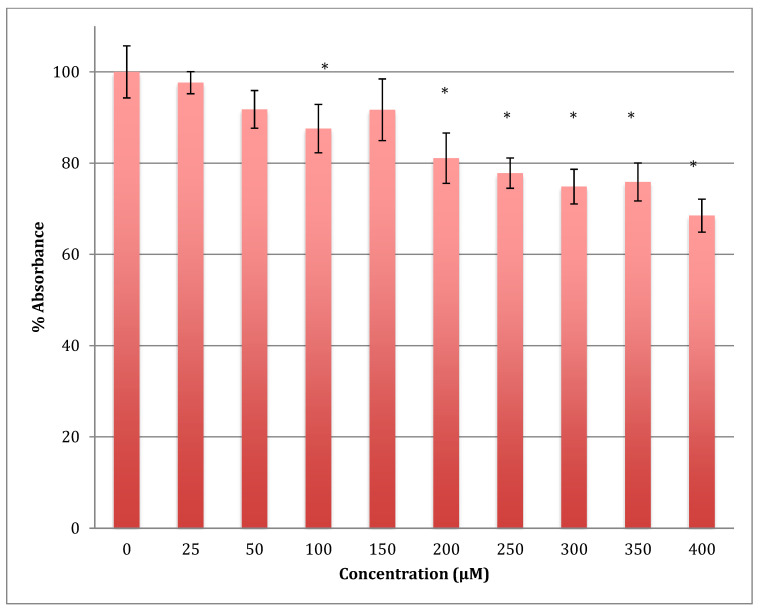
MTT assay measured in astrocytoma cells at 24 h. Results are percentage values (Mean ± SD, *n* = 3) where 100% corresponds to control values. Cells were treated with cobalt in a culture medium throughout the experiment. Data were analyzed by One-way ANOVA followed by a Tukey test. * Represents significantly different mean values between treatment and control. *p*-value < 0.05.

The MTT results showed a gradual decrease in the metabolic response of astrocytoma cells when treated with cobalt in a range of 0–400 μM for 24 h, reaching 68.5% of normal metabolism at the 400 μM cobalt concentration (Figure 4). The calculated LD_50_ was 713 ± 12 μM of cobalt. This indicates that cobalt is also a cytotoxic compound to the astrocytoma cells but less so than to SH-SY5Y cells.

**Figure 5:**
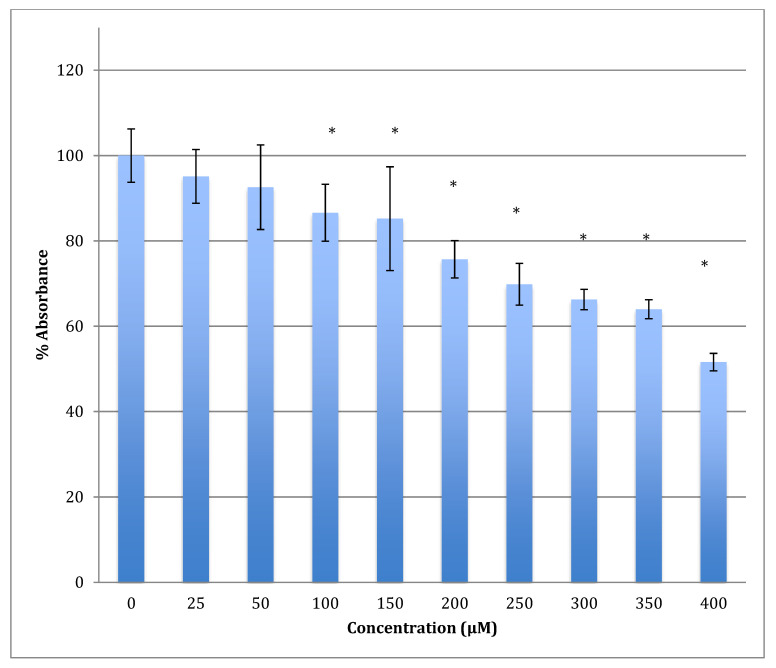
MTT assay measured in astrocytoma cells at 48 h. Results are percentage values (Mean ± SD, *n* = 3) where 100% corresponds to control values. Cells were treated with cobalt in a culture medium throughout the experiment. Data were analyzed by One-way ANOVA followed by a Tukey test. * Represents significantly different mean values between treatment and control. *p*-value < 0.05.

After 48 h of incubation with the same range of cobalt concentrations, the degree of decrease was steeper with a lower LD_50_ (422 ± 7 μM). For example, after treatment with 400 μM of cobalt, the metabolism of astrocytoma cells maintains only 51.6% viability when compared to non-treated cells (Figure 5). This indicates that cobalt is more harmful to the cells as the incubation period increases.

**Figure 6:**
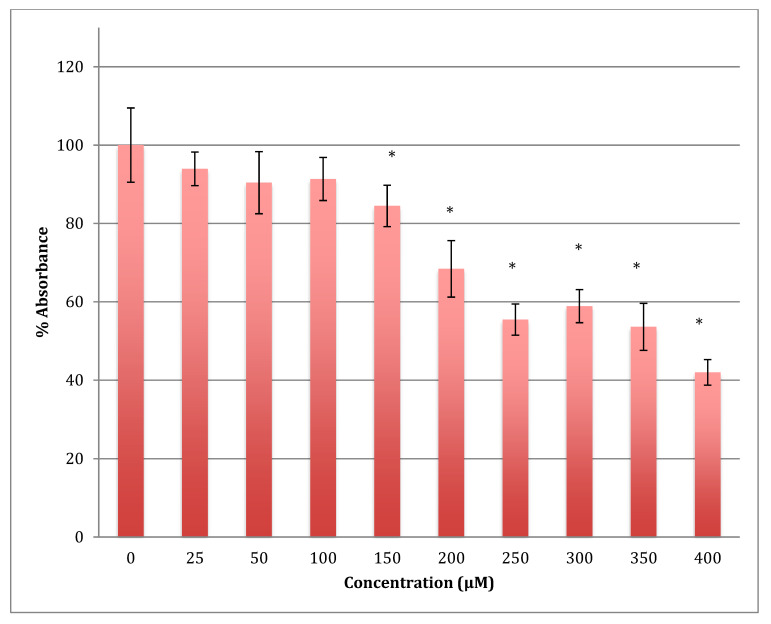
MTT assay measured in astrocytoma cells at 72 h. Results are percentage values (Mean ± SD, *n* = 3) where 100% corresponds to control values. Cells were treated with cobalt in a culture medium throughout the experiment. Data were analyzed by One-way ANOVA followed by a Tukey test. * Represents significantly different mean values between treatment and control. *p*-value < 0.05.

Similarly, after 72 h, the declining viability was even lower in astrocytoma cells when treated with the same range of concentrations of cobalt, reaching 42% viability at 400 μM. The calculated LD_50_ was 358 ± 8 μM (Figure 6). The degree of damage in astrocytoma cells at higher concentrations of cobalt with all incubation periods was less than that for the SH-SY5Y cells.

### 3.3. ROS Measurement in SH-SY5Y Cells after Treatment with Cobalt

**Figure 7:**
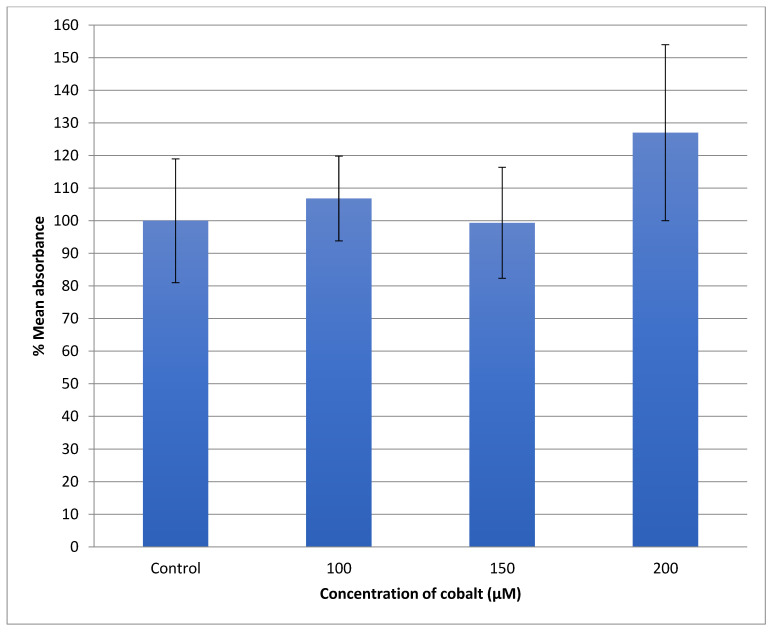
Measurement of ROS fluorescence intensity in SH-SY5Y cells upon exposure to 100, 150, and 200 μM of cobalt for 24 h, using carboxy-H2DCFDA to detect ROS at an excitation wavelength of 495 nm and emission wavelength of 525 nm. Data are Mean ± SD, *n* = 3, and were analyzed by One-way ANOVA (*p*-value < 0.05) followed by a Tukey test.

**Figure 8:**
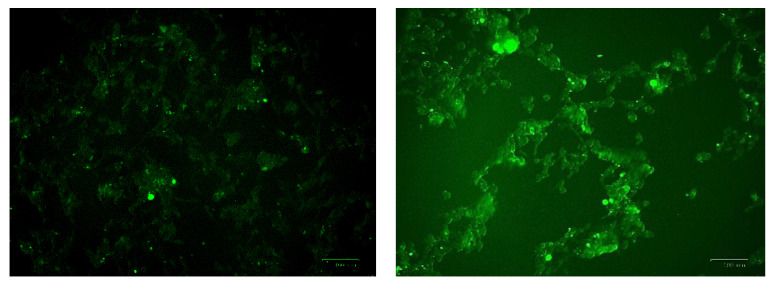
Images of SH-SY5Y neuroblastoma cells without cobalt treatment (on the top left) and after treatment with 100 μM of cobalt (on the top right). Green fluorescence indicates the amount of ROS production in the cells. Pictures were taken using the Motic AE31 microscope-100 power-dry lenses [Scale bar indicates 100 µm].

The generation of the reactive oxygen species (ROS) in SH-SY5Y cells after treatment with a range of cobalt concentrations (100, 150, and 200 μM) for 24 h was measured and compared with untreated controls as shown in Figure 7. Comparing fluorescence measurements of cobalt treated with non-treated SH-SY5Y cells for each incubation period, it was noticed that there was an insignificant increase in fluorescence in treated cells in comparison with controls after 4 h incubation with cobalt (data not shown as there was no fluorescence in all concentrations). On the other hand, there was an increase in the fluorescence measurement of the 200 μM treated cells after 24 h in comparison with the relevant controls (Figure 8). This is supported by the visual analysis of the dye concentration inside the cells, as the intensity increased as the concentration of cobalt increased. However, at the higher doses of cobalt (150 and 200 μM), more dead cells were floating in the medium which made the pictures not clear, and few cells were still attached to the base of the Petri dish. This finding shows that cobalt was increasing ROS production, especially at higher doses.

### 3.4. ROS Measurement in Astrocytoma Cells after Exposure to Cobalt

**Figure 9:**
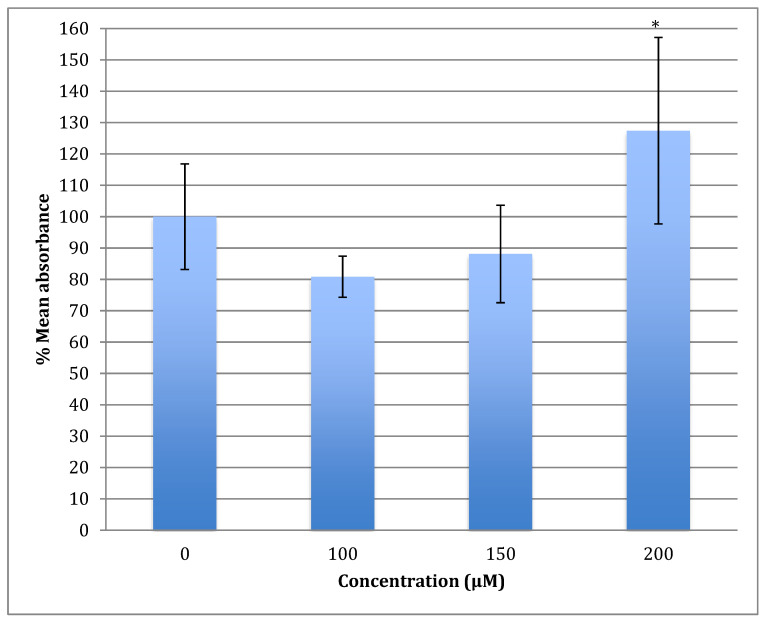
Measurement of ROS fluorescence intensity in U-373 cells upon exposure to 100, 150, and 200 μM of cobalt for 24 h, using carboxy-H2DCFDA at an excitation wavelength of 495 nm and emission wavelength of 525 nm for detection. Data are Mean ± SD, *n* = 3, and were analyzed by One-way ANOVA (*p*-value < 0.05) followed by a Tukey test. *: Indicate significant change compared to control.

**Figure 10:**
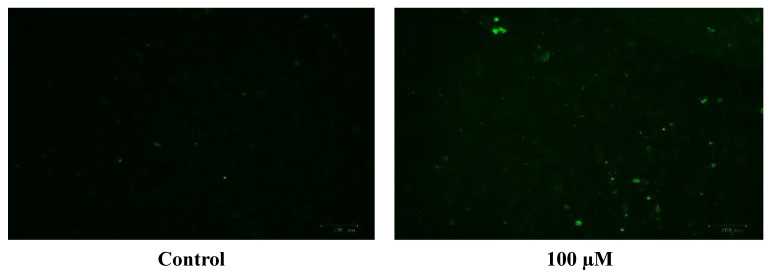

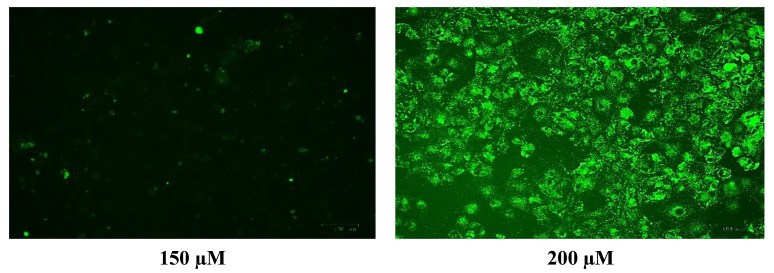
Images of U-373 astrocytoma cells without cobalt treatment (on the left), and after treatment with 100 μM of cobalt for 24 h (on the top right and the two pictures below). Green fluorescence indicates the amount of ROS production in the cells. Pictures were taken using the Motic AE31 microscope-100 power-dry lenses [Scale bar indicates 100 µm].

The U-373 cells were treated with a range of cobalt (100, 150, and 200 μM) for 24 h to measure the production of the reactive oxygen species (ROS) in comparison with non-treated cells and the results are shown in Figure 9. Comparing the fluorescence measurements of cobalt treated with non-treated U-373 cells for a 24-h incubation period, it was noticed that there was a significant (*p* < 0.05) rise in fluorescence in the control cells in contrast to a visible increase in fluorescence in the cobalt-treated cells at 200 μM concentration (Figure 10).

### 3.5. Quantitative Real-Time PCR for the Selected Genes for Neuroblastoma (SH-SY5Y) Cells

Quantitative real-time polymerase reaction (qRT-PCR) was carried out to test for the expression of specific genes after treatment of SH-SY5Y cells with a range of cobalt concentrations for 72 h. The genes were chosen to investigate if increased gene expression was linked to pathways involved in oxidative stress, DNA deamination, and DNA methylation. qRT-PCR for one reference gene RPL13A and five selected genes; Mlh1, SIRT2, MeCP2, UNG1, and TDG for SH-SY5Y cells after treatment with 25, 50, and 100 μM of cobalt was carried out. The gene expression in each treated sample and the gene expression in the non-treated sample (control) are shown in Table 2. The SIRT2 and MeCP2 gene expression was up-regulated at 50 and 100 μM of cobalt. All the other genes were less up-regulated when the cobalt concentrations increased.

### 3.6. Quantitative Real-Time PCR for the Selected Genes for the Astrocytoma Cells (U373)

Quantitative real-time PCR was carried out for one reference gene RPL13A and five selected genes; Mlh1, SIRT2, MeCP2, UNG1, and TDG for U-373 cells after treatment with 25, 50, and 100 μM of cobalt for 72 h. The selection of lower cobalt concentrations here was made to avoid the decline of cellular viability, causing all genes to be down-regulated. The gene expression was compared to the same gene expression in the non-treated cells as shown in Table 3.

There was highly significant up-regulation in the production of Mlh1 and SIRT2 genes at 25 μM cobalt, with no significant change in MECP2, UNG1, and TDG genes. Mlh1 and SIRT2 expression decreased at 50 μM to reach the same levels in the controls. However, at 100 μM concentration of cobalt, all genes were significantly up-regulated.

### 3.7. Metabolomic Study for Astrocytoma Cells

Data generated from LC/MS and analyzed by MzMine software were processed in Simca-P software to visualize the separation between control and treated astrocytoma samples.

**Figure 11:**
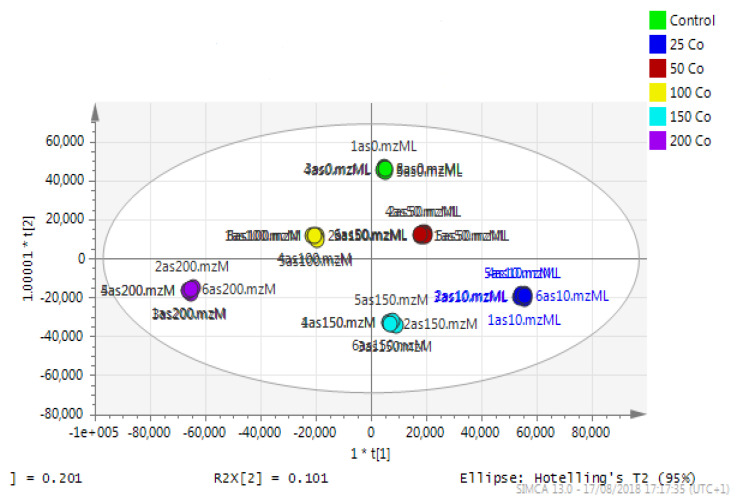
A PCA-X score plot of the separation between control and cobalt-treated U-373 cells after 72 h of incubation (*n* = 3). *: Indicating statistically significant change.

Astrocytoma cells showed reproducible data for within-sample replicates as there was almost no separation between replicates and no outliers. The separation was clear between groups (controls and cobalt-treated samples). The overall effect of cobalt on the astrocytes was demonstrated by a clear separation, using the unsupervised method of PCA, between cobalt-treated cells and the respective control cells (Figure 11). Cobalt-treated cells were shifted farther from the controls as the dose of cobalt increased. However, when a deeper analysis of the induced metabolic changes was carried out, it revealed significant changes in multiple metabolites (supplementary Table S1).

**Figure 12:**
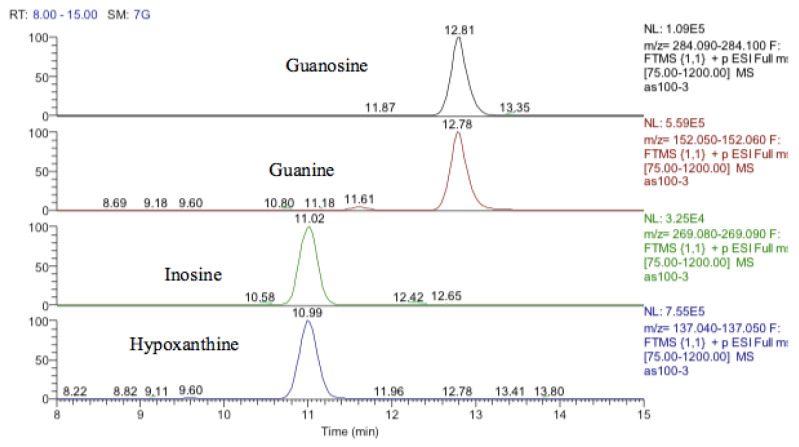
Extracted ion traces showing guanosine and a corresponding fragment for guanine and inosine and a corresponding fragment for hypoxanthine.

Inosine is an isomer of deoxyxanthosine, which is a potential oxidation product of deoxyguanosine. However, the retention time of the peak matched that of the inosine standard and, in addition, there was a clear fragment ion co-eluting corresponding to hypoxanthine (Figure 12). The same is the case for guanosine where 5-oxo deoxyguanosine, an important marker of DNA damage, is an isomer of guanosine. However, the compound which was elevated in response to treatment with cobalt was guanosine according to both its retention time and the co-eluting fragment for guanine (Figure 12).

**Figure 13:**
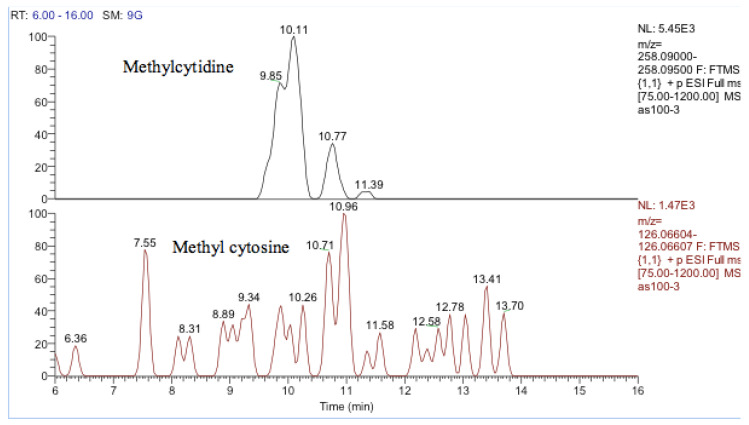
Extracted ion traces showing a weak peak for methylcytidine and a trace showing possibly traces of methyl cytosine.

One other nucleobase was elevated by treatment which was a methylguanosine. It matches the retention time of 1-methylguanine but in the absence of standards for other positional isomers, it was difficult to be confident in its identity. There was a very weak peak, which was elevated in the cells and had a mass corresponding to methylcytidine (Figure 13). Its retention time matched that of a standard for methyl cytidine. The elevation of MeCP2 protein resulting from treatment with cobalt indirectly suggests increased methylation of DNA and the most likely site for this is the five positions of cytidine.

There is some evidence for increased TCA cycle activity particularly at the higher doses of cobalt since there is a marked increase in the levels of NADH at 150 and 200 µM cobalt. In addition, the cobalt treatment elevated the levels of some acyl carnitines and CoA suggesting increased levels of fatty acid oxidation. Several partially oxidized fatty acids are elevated by the treatment.

### 3.8. Metabolomic Analysis of the Nuclear DNA for Astrocytoma Cells

It was not clear from the metabolomic profiling of the cell extracts if damage to DNA was a major effect of cobalt treatment. The DNA samples were injected into the LC–MS using the same metabolomics method. The LC–MS data yielded the results shown in supplementary Table S2. Again, it is not possible to determine much from these results since apart from deoxyadenosine the typical DNA bases are at low levels in the digest. Deoxyguanosine is present at about 10% of the level of deoxyadenosine, thymidine is absent, and deoxycytidine is only present in trace amounts. However, there appears to be methyldeoxycytidine and a large amount of hydroxymethyl deoxycytidine present in both the treated and control cells.

### 3.9. Metabolomics Results for the Neuroblastoma Cells

**Figure 14:**
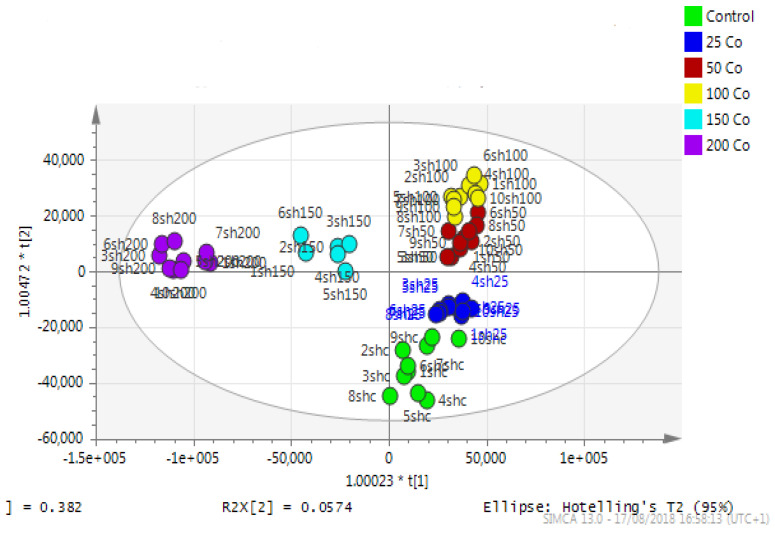
A PCA-X score plot of the separation between control and cobalt-treated SH-SY5Y cells after 72 h of incubation (n = 3). *: Indicating statistically significant change.

**Figure 15:**
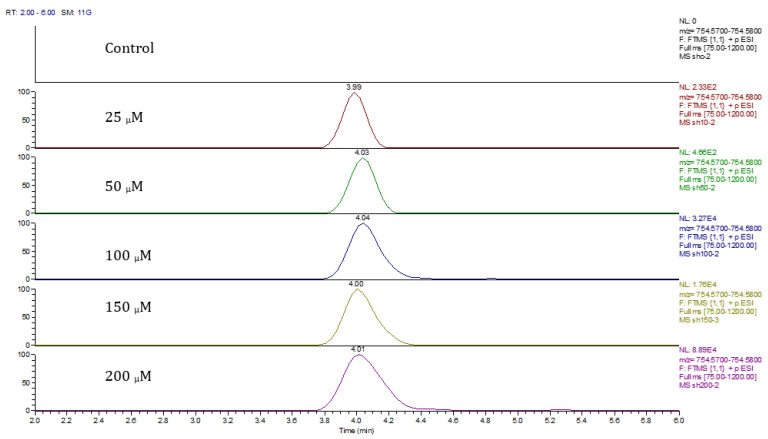
Extracted ion traces for PE 38:3 ether showing the effect of cobalt dose on its levels.

**Figure 16:**
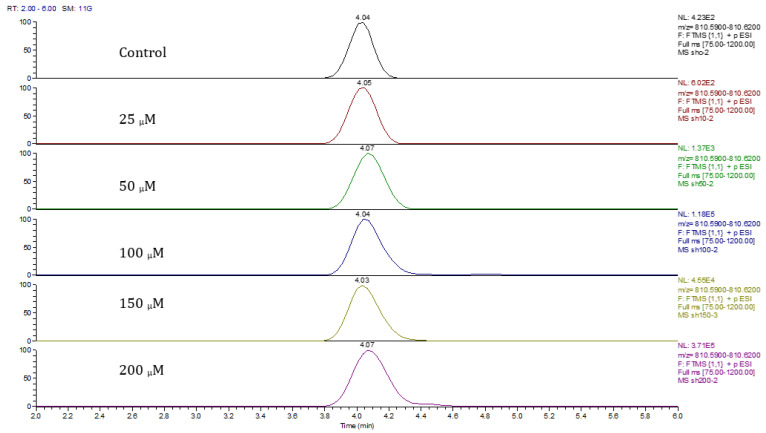
Extracted ion traces showing the effect of cobalt dose on the levels of PC38:4.

Principal component analysis (PCA) showed clear differences between control and cobalt-treated samples, and this became wider as the cobalt concentrations increased. The samples showed some variation within replicates but there were no outliers. Neuroblastoma cell data were analyzed using Simca-P to demonstrate geometrical separation and variation between treated and non-treated samples as illustrated in Figure 14. Supplementary Table S3 summarizes the changes in the metabolome of the neuroblastoma cells resulting from cobalt treatment. The effects on DNA and RNA bases are not as marked as in the case of astrocytoma cells. The RNA bases uridine, cytosine, and adenosine tend to be up-regulated but not consistently with dose. Again, the elevation of uridine and cytosine might be related to the formation of CMP choline and CMP ethanolamine for use in phospholipid biosynthesis. There is evidence of oxidative stress with glutathione and GSSG being elevated increasingly with cobalt dose. As with astrocytes, there is an effect on glycolysis with ATP being increased markedly with cobalt dose. However, the huge effect of cobalt is on some selected phospholipids, particularly ether lipids but also some acyl phospholipids. It is always important to check that the raw data reflects the extracted data particularly where changes are very marked, and Figure 15 shows a control sample where PE 38:3 is absent but is readily seen in the cobalt-treated samples and increases with dose. Similarly, Figure 16 shows the effect of cobalt dose on the levels of the acyl lipid PC 38:4 which increases about 1000-fold in response to the higher doses of cobalt. It is clear that cobalt stimulates major changes in the cell membrane of the SH-SY5Y cells, why it should stimulate this type of adaptation is not clear.

**Figure 17:**
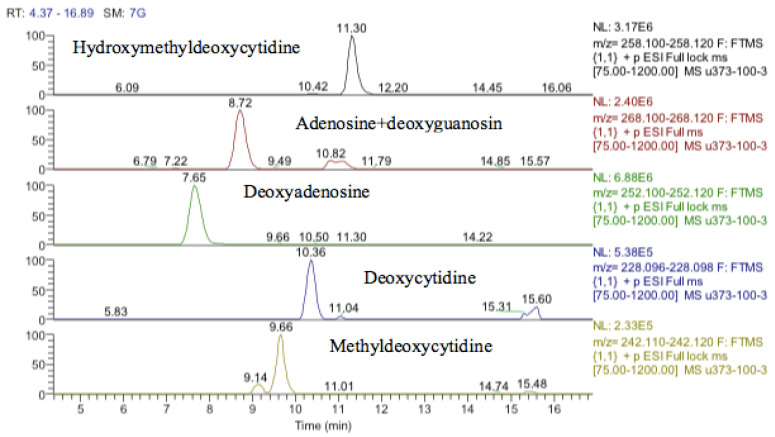
Extracted ion traces showing hydroxymethyldeoxycytidine, adenosine + deoxyguanosine, deoxyadenosine, deoxycytidine and methyldeoxycytidine.

**Figure 18:**
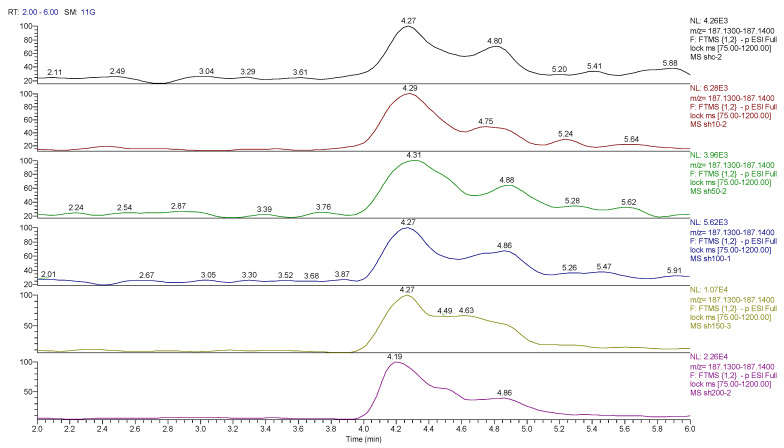
Extracted ion traces for hydroxy decanoic acid showing the effect of cobalt dose on its levels.

In addition to the effect of cobalt on phospholipids, it causes a general increase in levels of partially oxidized fatty acids. Many dioic acids are increased by the cobalt treatment. Figure 17 and Figure 18 show extracted ion traces for decandioic acid and its corresponding hydroxyl acid. Although the peak shapes are not good the elevation of the two compounds with cobalt dose is clear. From the analysis of the data obtained from the DNA digest, most of the bases decreased as the cobalt concentration increased except for uridine. However, cytidine triphosphate (CTP) increased significantly with the treatment of cobalt at all concentrations (25, 50, and 100 μM) after 72 h of incubation. The LC–MS data yielded the results shown in supplementary Table S4.

## 4. Discussion

Metal toxicity in humans has been widely known [21,22,23]. It is dose-dependent, at lower concentrations many metals are essential for life, and the same metals are toxic at higher doses.

Metabolomics has been widely used to investigate mechanisms of toxicology in human cells. We observed an increase in metabolism reaching a peak at 100 μM cobalt treatment in SH-SY5Y cells for 24 h. The increase in metabolism activity at lower concentrations of cobalt can be explained as a protective response of cells against cytotoxic cobalt compounds. This effect has been observed several times before in the literature, with diverse compounds, and is referred to as an adaptive response towards cell sensitization [24].

Several studies have reported the production of reactive oxygen species, especially hydroxyl radical (HO^•^), as the main toxic mechanism for metals to cause damage to proteins, lipids, and DNA in cells [25,26]. It is believed that astrocytes (astrocytoma) as they are located in the brain–blood barrier have a protective role around neurons and have higher antioxidation capacity than neurons [27]. Additionally, it is known that neurons rely on the astrocyte’s metabolic capacity to eradicate oxidative stress [28].

Quantitative real-time PCR of five selected genes; Mlh1, SIRT2, MeCP2, UNG1, and TDG showed fold change in expression compared to the reference gene. These genes were chosen to investigate if increased gene expression was linked to pathways involved in oxidative stress, DNA deamination, and DNA methylation could be detected as a result of cobalt treatment. Sirtuins (SIRTs) play an important role in the regulation of hypoxia-inducible factor-1α (HIF-1α). Under hypoxia, SIRT2 is the regulatory gene for HIF-1α transcription and stability [29]. Up-regulation of SIRT2 leads to a reduction in the production of HIF-1α. In contrast, down-regulation of SIRT2 yields an overexpression of HIF-1α [30]. SIRT2 was significantly increased as the cobalt dose increased suggesting that cobalt toxicity is mimicking the conditions produced by hypoxia and that SIRT2 is countering this. Cobalt has been proposed as a treatment for type II diabetes since it promotes the production of HIF1-α which is suppressed by hyperglycemia [31].

Methyl-CpG Binding Protein 2 (MeCP2) is predicted to bind to methylated promoters and silence transcription in SH-SY5Y cells [32]. MeCP2 has also been involved in nickel-induced gpt gene-silencing [33]. A decrease in MeCP2 levels was associated with the minimization of neuronal cell size [34]. In our results, it increased gradually as the concentration of cobalt increased.

Uracil DNA glycosylase gene (UNG1) is the major gene in base excision repair and the first to detect and repair mismatches in DNA. The increase (up to 2-fold at 100 μM) in UNG1 seen here indicates that base excision repair may play an important role in the defense mechanism of neuroblastoma cells against cobalt toxification. Mlh1 is one of the MutLgamma (MutLγ) genes (Mlh1 and Mlh3). The MutLgamma endonuclease causes R-loop-dependent CAG fragility. The observation of the up-regulation of Mlh1 provides evidence that breakage at expanded CAG repeats occurs due to R-loop formation and reveals a mechanism for CAG repeat instability mediated by cytosine deamination of DNA engaged in R-loops followed by up-regulation of other by MutLγ (Mlh1/Mlh3) producing cleavage [35]. The Mlh1 gene increased by 4-fold at 100 μM of cobalt compared to the relevant control. TDG is responsible for repairing methylation and the conversion of 5-methylcytosine to thymine. Thymine-DNA glycosylase (TDG) initiates base excision repair by cleaving the N-glycosidic bond between the sugar and target base [36]. This gene was up-regulated in the cells 2-fold at 100 μM of cobalt, compared to the control.

Mlh1 increased at 25 and 100 μM, which is responsible for CAG fragility and repeats. Thus, it is believed that cobalt at 100 μM concentration starts to induce harm to the structure of the nucleic acid. SIRT2 was significantly increased at 100 μM cobalt in the U-373 cells. An increase of SIRT2 reduces the production of HIF-1α as this suggests that in the case of these cells, cobalt is not inducing conditions mimicking hypoxia [37]. Interestingly, this gene increased in SH-SY5Y cells at all tested concentrations of cobalt. This provides evidence of the different functions and abilities of astrocytoma (astrocytes) as part of the blood–brain barrier, which protects neuronal cells against any toxification. SIRT2 is an important component in the antioxidant system and one of its functions is the activation of the pentose phosphate pathway to maintain levels of NADPH which can then be used to recycle GSSG back to GSH. The higher levels of SIRT2 in the astrocytes would be consistent with their improved resistance to cobalt-induced oxidative stress. Of all tissues, the brain has the highest levels of expression of SIRT2 [38]. Another increased gene was MeCP2, which was predicted, in a previous study [30], to bind to methylation promoters and silence transcription in SH-SY5Y cells. UNG1 and TDG were both up-regulated. They both play an important role in the base excision repair pathway. The up-regulation of these genes might be linked to the improved antioxidant defense of the astrocyte cells. It might be that higher concentrations of cobalt are required to see an effect on the expression of these genes in this cell line.

Comparing the two cell lines’ reactions to cobalt, they showed a different response to cobalt treatment. Production of genes associated with damage in cells increased at all concentrations of cobalt in the neuroblastoma cells. However, in astrocytoma cells the increase in gene expression was more abundant only at 100 μM of cobalt, indicating that these cells are more resistant to cobalt. This result suggests a different mechanism for defense against cobalt toxicity between the astrocytoma (representing the blood–brain barrier) and the neuroblastoma (representing neuronal cells in the brain).

Although it might be expected from the up-regulation of UNG and TDG1 that there would be evidence of base excision repair in the metabolomics data, there was no evidence for the presence of uracil which would be derived from oxidative damage of cytosine residues. However, there was one candidate marker for DNA excision repair, which was hydroxy thymidine. The database search had this compound listed as ribosyl imidazole acetate, which is a metabolite of histidine and an isomer of hydroxyl thymidine. Without a standard, it was difficult to be sure of the identity of this compound. However, there is a tendency for nucleosides to fragment in the source of the instrument losing the sugar portion of the molecule. Thus, evidence of the identity of hydroxy thymidine is provided by a fragment of hydroxy thymidine, hydroxy methyl uracil, which elutes at the same time as the putatively identified hydroxy thymidine. It is known that the oxidation of either 5-methylcytosine or thymine will end with the formation of 5-hydroxymethyluracil [39]. However, in the current case, there is no evidence of the presence of hydroxy methyl uracil other than the fragment formed in the instrument from hydroxymethylthymidine. Hydroxymethylthymidine has not been reported as a DNA excision product.

5-Methylcytosine can be spontaneously deaminated and this reaction will result in the formation of thymine and ammonia, and this deamination is the predominant observed single transmutation. If this mutation was detected in the DNA before the next replication, it would be fixed by the thymine-DNA glycosylase (TDG) enzyme, which detaches thymine in the mismatching of G-T. This creates a basic location in the DNA which will be corrected by AP endonucleases, for example UNG1 [40]. Cytosine can be methylated to 5-Methylcytosine when a methyl group is linked to carbon 5, which will change the structure but not the base pair function [41]. The formation of 5-Methylcytosine is an external genetic alteration caused by DNA methyltransferases [42]. 5-methylcytosine was detected in both cell lines in this experiment and an isomer of thymine was also detected but the peak matching thymine, which had changed significantly, had a retention time of 11.4 min and thus was an isomer of thymine. The peak for the thymine standard runs at 7.3 min and the peak corresponding to this retention in the samples was not changed by treatment. There were strong effects on guanosine and inosine with both these metabolites being highly elevated by treatment.

S-adenosyl methionine (SAM) is the main agent for methylation in biological systems and is elevated in response to cobalt treatment as is S-adenosylhomocysteine which is the product resulting from the transfer of a methyl group. Possibly linked to this are elevated levels of trimethylysine. Trimethylysine is generated from the degradation of histone proteins, which are methylated by SAM. It has been found that MeCP2 promotes methylation of histone tails which is part of its gene-silencing activity since methylated histones bind more tightly to DNA [43]. The turnover of histones could lead to an increase in the levels of trimethyl lysine. Trimethyllysine is the biosynthetic precursor of trimethylaminobutanoate, which is hydroxylated to form carnitine and both compounds are elevated by treatment with cobalt.

FDR (False discovery rate) and adjusted *p*-value are statistical measures used in hypothesis testing to control multiple testing. The *p*-value is the probability of obtaining a result as extreme or more extreme than the observed result under the assumption that the null hypothesis is true. A *p*-value is considered statistically significant if it is less than a predetermined threshold, often 0.05. However, when multiple tests are performed simultaneously, the probability of obtaining a significant result by chance alone increases, and the probability of making a Type I error (rejecting the null hypothesis when it is actually true) also increases.

To address this issue, adjustment methods such as the Bonferroni correction, Holm’s method, and the Benjamini–Hochberg method are used. These methods adjust the *p*-value threshold to account for the number of tests being performed, to control the family-wise error rate (FWER) or the false discovery rate (FDR). FDR is the expected proportion of false positives among all the significant results, and it controls the expected proportion of Type I errors among all significant results. The FDR is typically less stringent than the FWER, which makes it more sensitive and allows the detection of more true positives. The Benjamini–Hochberg method is a widely used method for controlling the FDR.

When obtaining metabolites with false discovery rate (FDR)-adjusted *p*-values (<0.05), we have obtained 21/797, 128/797, 219/800, 43/798, and 42/797 metabolites in human astrocytoma cells (U373) treated with 25 μM, 50 μM, 100 μM, 150 μM, and 200 μM cobalt concentration (Supplementary Table S5). Similarly, FDR-adjusted *p*-values (<0.05), showed 27/1644, 131/1637, 283/1587, 276/1538, and 412/1556 metabolites in SH-SY5Y cell lines at the same cobalt concentration (Supplementary Table S6).

The astrocytoma cells show marked effects on the nucleosides cytosine and uridine which accumulated increasingly as the dose of cobalt increased. These are RNA bases rather than DNA bases. The effects on uridine appear to be particularly significant since UDP and UTP are elevated as well as UDP-glucose. Uridine can be readily converted to cytidine and CMP and CTP are also increased by cobalt treatment [44]. The increase in UDP-glucose suggests that cobalt may be stimulating glycogen biosynthesis since UDP-glucose is the precursor for glycogen formation. UDP-glucose is formed from UTP and glucose phosphate, both of which are increased by cobalt treatment. However, UDP-xylose is also increased and there are non-significant but persistent increases in UDP-N-acetylglucosamine (UNAC) with cobalt dose, linked to this is the accumulation of the N-acetylglucosamine phosphate which is a precursor of UNAC. This suggests there might be some effect on the biosynthesis of glycan chains which are attached to glycoproteins, and this might relate to the observation that the astrocytes lose their ability to adhere to the culture dish following cobalt treatment since glycoproteins are involved in cell adhesion.

CDP-choline and CDP-ethanolamine are both required for phospholipid biosynthesis. The are several phospholipids that are significantly increased in the treated cells and the effect is most marked for the cells treated with 25 µM cobalt suggesting that the capacity of the astrocytes to respond to cell membrane damage is greatest at the lowest concentration of cobalt. The clearest effect of cobalt in astrocytes is in stimulating the antioxidant defense system and this might be expected from the observation that SIRT-2 is highly up-regulated in the cells. Glutathione disulfide is an oxidized form of the thiol antioxidant glutathione. It is a disulfide formed from two glutathione molecules [45], and can be formed as a result of the reduction of peroxides such as H_2_O_2_ and ROOH [46]. Increased glutathione disulfide is considered an important marker for oxidative stress, and has been detected in neurodegenerative diseases [47]. Glutathione disulfide decreased after exposure to lower concentrations of cobalt (at 25 and 50 μM) for 72 h in neuroblastoma cells, but it increased gradually at higher concentrations of cobalt (100, 150, and 200 μM). This might be explained by the lower ability of neuroblastoma cells to respond to oxidative stress as indicated by the lower LD_50_ value obtained in comparison with astrocytes. However, in astrocytoma cells, there was a gradual increase in the level of glutathione disulfide from 2.85 (at 25 μM of cobalt) to 9.75 (at 200 μM of cobalt). Astrocytoma cells have a better oxidative defense mechanism than neuroblastoma cells as part of their function as a component of the blood–brain barrier requires [48].

**Figure 19:**
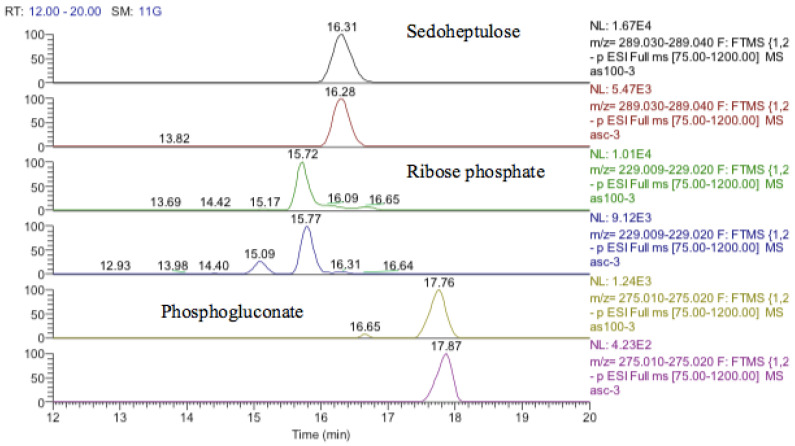
Extracted ion traces for increased pentose phosphate pathway metabolites, sedoheptulose, ribose phosphate, and phosphogluconate.

The antioxidant defenses in the astrocytoma cells are strong since GSH levels are maintained although the GSSG levels also increase markedly. This strong antioxidant defense is related to the increase in SIRT-2 discussed above. The pentose phosphate pathway is up-regulated since sedoheptulose phosphate, 6-phosphogluconate, and ribose phosphate are increased (Figure 19) and the diversion of glucose into this pathway provides one of the major routes for converting NADP+ into NADPH. NADPH is required for recycling GSSG recycling back into GSH via glutathione reductase. There is some evidence for increased glycolysis in response to cobalt treatment since glucose1-phosphate, glucose 5-phosphate, glyceraldehyde phosphate, and fructose bisphosphate, which are all in the glycolytic pathway, are all increased. This is reflected to some extent in the slightly increased levels of ATP in response to treatment. The use of cobalt to stimulate glycolysis is supported by these data but an increase in glycolysis contradicts the increased levels of SIRT-2 which is supposed to correlate with higher levels of HIF1-α which should promote oxidative phosphorylation and an increase in fatty acid oxidation and the TCA cycle.

The hydroxymethyl deoxycytidine is isomeric with the RNA base 5-methylcytidine but the standard for 5-methylcytidine runs 5 min earlier than the putatively identified hydroxymethyldeoxycytidine. However, such modifications are rare in DNA, and it would be unusual to see such a high level of modification of the DNA thus it is possible that the hydroxymethyldeoxycytidine is an RNA base that has been methylated within the ribose portion of the molecule. It is difficult to decide on what these data mean particularly since the methyl cytosines are high in the controls as well as the treated cells. Metabolomic profiling of purified DNA has only been carried out rarely and in view of the somewhat unusual findings it would be necessary to repeat this experiment to gain firm conclusions.

Many dioic acids are increased by the cobalt treatment. Dioic acids are produced by the peroxisomal oxidation of long-chain fatty acids via hydroxylation in position 1 of the fatty acid chain [49]. Without a suitable standard, it is not possible to specify the position of hydroxylation in the current data, but many hydroxy fatty acids are also elevated. CTP is a substrate used to synthesize RNA [50] and it is not clear why CTP increased here since it should not be a part of DNA, and phosphatase treatment used during the isolation should have converted it to cytidine. Overall, it is not possible to determine much from these data and the digest procedure likely failed to properly digest the DNA into its constituent nucleosides.

## 5. Limitation of This Study

The major limitation of the work reported in this paper is the extrapolation from cells cultured in a Petri dish to cells living in the in vivo environment in the brain and furthermore to the effects on the whole human body.

## 6. Summary

1. The results of the MTT assay have shown that cobalt is toxic to the SH-SY5Y and U-373 cells with more potency in SH-SY5Y cells.
2. ROS measurement assay showed an increase in fluorescence in SH-SY5Y cells at 100 μM of cobalt and in U-373 cells at 200 μM of cobalt.
3. Metabolomic analysis of both cell lines (SH-SY5Y and U-373) showed that cobalt is inducing an intracellular change in both cell lines.
4. The metabolomic analysis of whole cell extracts showed an increase in metabolites associated with oxidative stress and glutathione oxidation pathways. The evidence for DNA methylation and hydroxymethylation was weaker.
5. The metabolomics analysis of the extracted DNA of both cells did not show conclusive changes in the levels of modified DNA. The lack of clear results might be due to the very low levels of this type of modification within DNA, the level of RNA contamination of the DNA sample, and possibly incomplete digestion of the sample.
6. The results of the RT-PCR experiment showed an increase in the genes (Mlh1, SERT2, MeCP2, UNG1, and TDG) in both cell lines after treatment with cobalt, and these genes were associated with the aforementioned pathways in both cell lines.

## 7. Conclusions

In the current study, neuroblastoma and astrocytoma cells were treated with a range of cobalt concentrations for different periods to investigate the effects of cobalt on the metabolism of human neuronal-derived cells. Proliferative methods combined with metabolomics and real-time PCR were applied in the investigation. Results provided tentative proof of DNA deamination, DNA methylation, and glutathione oxidation. It is believed that oxidative stress is the main effect that triggers changes in the other pathways.

## Figures and Tables

**Table 1:** Six primer sets (forward and reverse) were designed and used for gene quantification in the RT-PCR experiment.

Genes		Primer Sequences (5′->3′)	Tm	Amplicon Size (bp)
SERT2	Forward	GAA GGT GCA GGA GGC TCA G	60.08	149
	Reverse	CCA AGG TCA GCT CGT CCA G	60.08	
UNG	Forward	ACC TGG ACC CAG ATG TGT GA	60.47	144
	Reverse	ATA AAT GTT CTC CAA ACT GGG CG	59.56	
TDG	Forward	CAT TGT CAT TAT TGG CAT AAA CCC G	59.31	150
	Reverse	CCT GGT AGA GTG TGA TCA TCC AT	59.35	
MLH1	Forward	TTT CGA GGT GAG GCT TTG GC	60.89	149
	Reverse	GTC CCT TGA TTG CCA GCA CA	60.90	
MECP2	Forward	TGA TCA ATC CCC AGG GAA AAG C	60.62	150
	Reverse	TAG GTG GTT TCT GCT CTC GC	59.75	
RPL13A	Forward	ACC TCC TCC TTT TCC AAG CG	59.96	141
	Reverse	GCG TAC GAC CAC CAC CTT C	60.74	
HPRT1	Forward	CTG GAA AGA ATG TCT TGA TTG TGG A	59.53	135
	Reverse	TTC GTG GGG TCC TTT TCA CC	60.18	
B2M	Forward	GTG CTC GCG CTA CTC TCT C	60.30	136
	Reverse	CGG ATG GAT GAA ACC CAG ACA	60.07	

**Table 2:** Gene transcription fold changes in SH-SY5Y cells after treatment with a range of cobalt concentrations. Fold change values are represented as mean values ± (STD).

	Cobalt Concentration (μM)				
Gene		Control	25	50	100
Mlh1	Fold change	1 ± (0.14)	0.79 ± (0.15)	2.15 ± (0.11)	4.30 ± (0.05)
	ΔΔC_T_	0	0.34	−1.10	−2.11
SIRT2	Fold change	1 ± (0.26)	2.88 ± (0.28)	9.87 ± (0.12)	17.60 ± (0.09)
	ΔΔC_T_	0	−1.53	−3.30	−4.14
MeCP2	Fold change	1 ± (0.23)	2.31 ± (0.33)	11.04 ± (0.24)	24.11 ± (0.14)
	ΔΔC_T_	0	−1.21	−3.46	−4.59
UNG1	Fold change	1 ± (0.07)	0.39 ± (0.02)	1.40 ± (0.03)	2.34 ± (0.07)
	ΔΔC_T_	0	1.37	−0.49	−1.22
TDG	Fold change	1 ± (0.22)	0.85 ± (0.19)	1.39 ± (0.29)	2.13 ± (0.13)
	ΔΔC_T_	0	0.24	−0.48	−1.09

**Table 3:** Gene expression exhibited as fold changes in U-373 cells after treatment with a range of cobalt concentrations. Fold change values represented as mean values ± (STD).

		Cobalt Concentration (μM)			
Gene		Control	25	50	100
MLH1	Fold change	1 ± (0.15)	1.67 ± (0.23)	0.92 ± (0.18)	7.89 ± (0.06)
	ΔΔC_T_	-	−0.74	0.12	−2.98
SIRT2	Fold change	1 ± (0.27)	2.89 ± (0.24)	1.13 ± (0.28)	7.68 ± (0.28)
	ΔΔC_T_	-	−1.53	−0.18	−2.94
MeCP2	Fold change	1 ± (0.19)	1.23 ± (0.36)	0.37 ± (0.31)	4.93 ± (0.36)
	ΔΔC_T_	-	−0.30	1.45	−2.30
UNG1	Fold change	1 ± (0.16)	1.27 ± (0.22)	0.66 ± (0.04)	5.99 ± (0.04)
	ΔΔC_T_	-	−0.35	0.61	−2.58
TDG	Fold change	1 ± (0.17)	0.86 ± (0.25)	0.43 ± (0.05)	3.61 ± (0.05)
	ΔΔC_T_	-	0.21	1.23	−1.85

## Data Availability

The original contributions presented in the study are included in the article/Supplementary Material. Further inquiries can be directed to the corresponding authors.

## Supplementary Materials

The following supporting information can be downloaded at: https://www.mdpi.com/article/10.3390/metabo13060698/s1, Table S1: Metabolomic changes in U-373 cells resulting from cobalt treatment; Table S2: Metabolomic data derived from LC-MS of the nuclear DNA after separation from astrocytoma treated with cobalt at 25, 50 and 100 μM and non-treated for 72 h Table S3: Induced changes in SH-SY5Y cells after treatment with 0-200 μM of Co for 72 h. Table S4: Metabolomic data derived from LC-MS of the nuclear DNA after separation from neuroblastoma treated (25, 50 and 100 μM) and non-treated with cobalt for 72 h Table S5: False discovery rate (FDR)-adjusted *p*-values (U373), Table S6: False discovery rate (FDR)-adjusted *p*-values (SH-SY5Y).
